# “It's a burden, it's a nuisance. I wish I didn't have these other ailments”: a qualitative exploration of comorbidities management among older people living with HIV who use drugs in Vancouver, British Columbia

**DOI:** 10.1002/jia2.25785

**Published:** 2021-10-11

**Authors:** Koharu Loulou Chayama, Cara Ng, Will Small, Andrew Ivsins, Ryan McNeil

**Affiliations:** ^1^ British Columbia Centre on Substance Use Vancouver British Columbia Canada; ^2^ Department of Medicine University of British Columbia Vancouver British Columbia Canada; ^3^ Faculty of Health Sciences Simon Fraser University Burnaby British Columbia Canada; ^4^ Department of Internal Medicine Yale School of Medicine New Haven Connecticut USA; ^5^ Program in Addiction Medicine Yale School of Medicine New Haven Connecticut USA

**Keywords:** ageing, comorbidities, drug use, HIV

## Abstract

**Introduction:**

People living with HIV (PLHIV) who use illicit drugs (other than or in addition to cannabis) are living longer due to antiretroviral therapy (ART). Older PLHIV who use drugs have an increased risk for comorbidities, and managing multiple health conditions is a growing concern among this population. However, in‐depth understandings of the lived realities and complexities of living with HIV alongside comorbidities among older PLHIV who use drugs remain limited. We sought to explore how older PLHIV who use drugs manage their comorbid conditions in a setting with universal ART access.

**Methods:**

Between January 2019 and March 2020, semi‐structured, in‐depth interviews were conducted in Vancouver, Canada with 42 older PLHIV who use drugs and were living with at least one comorbidity. All participants were currently on ART, and had initiated treatment at least 2 years prior to the interviews. Data were analysed using inductive and deductive approaches.

**Results:**

Several themes were identified through this analysis. First, comorbidities were perceived as more urgent health concerns and prioritized over HIV. Second, stigma and discrimination hindered access to care for comorbidities. Third, the concurrent management of HIV and comorbidities was often challenging due to unmanaged or poorly managed comorbidities. Fourth, the potential impact of ART on the development of comorbidities was a source of concern and frustration. Finally, integrated treatment approaches facilitated engagement with HIV and comorbidities care.

**Conclusions:**

Our findings underscore the need for HIV care to shift from a primary focus on managing HIV to an integrated, patient‐centred approach that addresses both HIV and non‐HIV‐related health needs, as well as an equitable and non‐judgemental delivery of such care for an ageing population of PLHIV who use drugs.

## 1 INTRODUCTION

Globally, 5.7 million people living with HIV (PLHIV) are 50 years of age or older [1]. Since the development of antiretroviral therapy (ART) in the mid‐1990s and its widespread adoption, life expectancy has increased among PLHIV and their morbidity and mortality patterns have shifted from acute HIV‐related opportunistic infections to non‐HIV‐related chronic diseases [2, 3]. While comorbid chronic conditions can develop at any age, the burden of comorbidities increases in older age [4]. Higher comorbidity burden among PLHIV has been associated with suboptimal HIV outcomes, including lower CD4 cell counts and higher viral load, and lower health‐related quality of life [4, 5, 6]. As the ageing population of PLHIV continues to increase across the world, there have been growing calls for policy and research attention to comorbidity management as part of HIV care [7, 8].

PLHIV are at elevated risk for a range of chronic diseases, including type 2 diabetes, non‐AIDS defining cancers and renal, pulmonary and cardiovascular diseases [9, 10, 11, 12, 13]. Several possible explanations for the high prevalence of chronic diseases in PLHIV have been identified. Chronic diseases in PLHIV may be attributed to long‐term inflammation from HIV and adverse side effects from ART [14, 15]. In addition to biological and individual factors, there has been a growing recognition that social (e.g. stigma) and structural conditions (e.g. poverty) create and perpetuate vulnerabilities placing PLHIV at a higher risk for comorbidities [16]. Research has provided compelling evidence for the important role of the “risk environment” and elucidated how contextual factors produce harm for PLHIV who use illicit drugs (hereafter referred to as PLHIV who use drugs) [17, 18]. The risk environment as a conceptual framework refers to the interplay between environments (i.e. physical, social, economic and policy) operating across the micro‐ (i.e. immediate), meso‐ (i.e. institutional) and macro (i.e. societal) levels that shape drug‐related harm [17, 18]. Prior work has documented high prevalence of comorbidities among PLHIV who use drugs [19]. Compared to their non‐drug‐using counterparts, PLHIV who use drugs are placed at higher risk for a variety of diseases and illnesses, including substance use disorder, viral hepatitis, tuberculosis, bacterial infections, mental health disorders and chronic pain, resulting in an increased burden of morbidity and mortality [19, 20]. Management of comorbidities in PLHIV who use drugs complicates care and introduces a range of challenges, such as drug–drug interactions and adherence to HIV and non‐HIV treatments that must be appropriately addressed [19].

However, in‐depth understandings of the lived realities and complexities of living with comorbidities among PLHIV who use drugs remain limited, especially among the ageing population. No literature to date has examined how older PLHIV who use drugs manage their multiple health conditions and overlapping needs within the context of social‐structural inequalities. We undertook this study to explore the experiences of managing HIV alongside comorbidities among older PLHIV who use drugs and how these are shaped by social‐structural forces operating within the risk environment. Greater understanding of how older PLHIV who use drugs manage their HIV and comorbidities will be essential to informing efforts to optimize care strategies and improve the health and quality of life of this population.

## 2 METHODS

We draw upon 42 semi‐structured, qualitative interviews conducted with older PLHIV who use drugs (other than or in addition to cannabis) and were living with at least one comorbidity (see demographics in Table 1). The study was conducted in Vancouver, Canada, a setting with universal ART access, and all participants were on ART. Given the limited research in this area, qualitative interviews are particularly well positioned to generate initial insights into how this population manages their comorbidities and guide future research. The study was approved by the University of British Columbia/Providence Health Care Research Ethics Board.

**Table 1:** Background characteristics of sample (*n* = 42)

	Total	(%)
**Age**		
50–59	30	(71)
60–69	12	(29)
**Gender**		
Man	24	(57)
Woman	17	(41)
Other	1	(2)
**Race/ethnicity** ^a^		
Asian	2	(5)
Black	1	(2)
Indigenous	16	(40)
White	26	(62)
**Drug use in past 30 days** ^a^		
Amphetamine	24	(57)
Cocaine	34	(81)
Crystal methamphetamine	17	(41)
Opioids (heroin/fentanyl)	19	(45)
Non‐medical prescription opioid use	14	(33)
Ritalin	12	(31)
Other	16	(40)
**Comorbidities** ^a^		
Chronic pain	27	(64)
Chronic obstructive pulmonary disease (COPD)	12	(31)
Cardiovascular disease	10	(24)
Diabetes	2	(5)
Hepatitis C	28	(67)
Mental illness	17	(41)
Opioid use disorder	11	(26)
Stimulant use disorder	10	(24)
Other	21	(50)

^a^Participants could report multiple categories.

### 2.1 Participant recruitment

PLHIV were eligible for the study if they were: 50 years and older; used illicit drugs other than cannabis in the last 30 days; initiated ART at least 2 years prior to study entry (regardless of a history of treatment interruptions); and diagnosed with at least one comorbidity. Participants were recruited using two approaches to enhance the diversity of the study sample. First, participants were recruited through outreach at the Dr. Peter Centre, an integrated HIV care facility serving PLHIV who use drugs in Vancouver's West End neighbourhood through its low‐barrier day health program and specialized nursing care residence. A recruitment booth was set up at the facility to provide information about the study and centre clients were invited to participate. Those who expressed interest in participating were then screened for eligibility and, if eligible, scheduled an interview. Second, participants were recruited from a cohort of individuals enrolled in the AIDS Care Cohort to evaluate Exposure to Survival Services (ACCESS), a community‐recruited open prospective cohort study of PLHIV who use drugs. Participants were eligible for ACCESS if they were 18 years of age or older, lived in Greater Vancouver and used illicit drugs other than cannabis in the previous month (21). ACCESS participants were recruited through word of mouth, street outreach and referrals [21]. For the current study, ACCESS participants were screened for eligibility during their routine cohort study interviews. Those eligible and interested in participating were scheduled for an interview. Recruitment concluded with 42 participants, upon reaching saturation in data collection and a sufficiently diverse sample in relation to gender and race/ethnicity.

### 2.2 Data collection

Interviews were conducted by two masters‐qualified and experienced qualitative research coordinators (KLC and CN) in a private room at the Dr. Peter Centre or BCCSU field office. Prior to each interview, interviewers provided participants with an overview of the study, answered any questions and obtained written informed consent. A survey was used to collect demographic information (e.g. drug use patterns and HIV treatment history) and an interview guide was used to facilitate discussion regarding how participants managed their HIV and comorbidities. The interview guide was developed based on a review of the relevant literature and by drawing on the research team members’ expertise conducting qualitative research with PLHIV who use drugs. Interviews lasted approximately 60 min and were audiorecorded. Participants received an honorarium ($30 CAD) as compensation for their time. Interviews were conducted between January 2019 and March 2020. Interviews were transcribed by a professional transcription service and reviewed for accuracy by the interviewers. An online random name generator was used to give study participants pseudonyms.

### 2.3 Data analysis

Interview transcripts were imported into NVivo qualitative analysis software to facilitate coding and thematic extraction using both inductive and deductive methods. Following an inductive approach, data analysis started during data collection for emergent themes to be incorporated into subsequent data collection. The research team developed a preliminary coding framework after approximately 25 interviews. Codes were developed based on deductive *a priori* themes derived from the interview guide, literature review, expertise of the research team and preliminary themes emerging from the initial interviews (e.g. “HIV management”, “comorbidity management” and “health priority”). The data were then coded by two members of the research team. During data collection and coding, the research team met regularly to discuss new themes that emerged and refined the coding framework to fully account for participant experiences. To interpret our themes, we then drew upon the Risk Environment Framework. Through comparison to narrative data, we sought to characterize how the participants’ experiences of managing HIV and comorbidities were shaped by contextual forces. Of particular interest were how features of the risk environment shaped access to and engagement with treatment and care for their multiple health conditions.

## 3 RESULTS

Five themes emerged from data analysis. First, comorbidities were prioritized over HIV. Second, stigma and discrimination hindered access to comorbidities care. Third, concurrent management of HIV and comorbidities was often challenging to juggle. Fourth, the potential impact of ART on the development of comorbidities was a source of concern and frustration. Finally, integrated treatment approaches facilitated engagement with HIV and comorbidities care.

### 3.1 Prioritizing comorbidities over HIV

The majority of participants explained that HIV was no longer their most pressing health concern, as many had been diagnosed with HIV in the 1990s or early 2000s and had grown to view it as a manageable chronic disease. These participants reported that, while managing HIV was “obviously” important, their HIV‐related health needs were under control when they were adherent to ART. Most of our participants had initiated treatment during the early era of ART – characterized by pill burden and side effects (e.g. nausea and fatigue) often resulting in suboptimal treatment adherence and in turn poor outcomes [22]. In contrast, participants reported improvements to their ART regimens (e.g. fewer pills and side effects) following advances in HIV treatment, leading to better treatment tolerability and, in many cases, HIV viral suppression. As “Dan”, a 55‐year‐old white man who had been living with HIV for 30 years, described:

Dan: For the first 15 years, I had zero T cells, and now my helper cells are around 650 and I have been undetectable for the last 15 years.
Interviewer: What was going on before that when you didn't have undetectable viral loads?
Dan: It [HIV treatment] wasn't working out much. When I was prescribed AZT, I had chronic pill fatigue and was tired of taking meds so I just stopped.

Participants explained how comorbidities often took priority over HIV, as management of these conditions was more challenging. Given their medical complexity and older age, participants described lack of access to adequate treatment for their comorbidities. For example, for “Lindy”, a 57‐year‐old Indigenous woman living with COPD, her lung health took priority over other health issues. Despite experiencing significant difficulties breathing and engaging in daily activities, she had not been able to receive a diagnosis and therefore appropriate treatment for her condition:

Interviewer: What is your biggest health concern or priority for you right now?
Lindy: My lungs are what really, really scares me. […] This winter, it was so hard to breathe. […] That's the one thing that I'm more concerned about. I'm not really concerned about anything else. I get concerned about my liver and kidneys and whatever, right, because I know they're all going to go sooner or later. But I know it's my lungs right now that are in really bad shape. I mean when they tell you you have to see a specialist, and they can't figure what's going on, that is scary. That's really scary when I can't walk that far without going [panting for breath].

For, “Jamie”, a 55‐year‐old white man with a kidney failure, concerns about his current dialysis treatment were paramount as he frequently experienced severe post‐dialysis symptoms. However, his older age and multimorbidity hindered his ability to access a kidney transplant, leaving him with no other option but to continue dialysis:

Well, I worry more about my dialysis than I worry about the HIV. The HIV I'm not going to die from. I'm going to die from dialysis or maybe a stroke. When I come from dialysis, I feel like I'm having multiple strokes. […] I'm worried about dying from dialysis because they're not going to give me a kidney transplant. I'm too old and I got too many things wrong with me.

### 3.2 Stigma and discrimination as barriers to care for comorbidities

Participants expressed that even as they had gained access to HIV care through HIV‐specific providers and facilities, stigma and discrimination in health care encounters impeded their access to comorbidities care. Participants reported living with a wide range of non‐HIV health conditions, with the most common being chronic pain, hepatitis C, COPD, cardiovascular disease, mental illness and substance use disorder. Some participants described experiencing anti‐drug stigma from health care providers, even when receiving treatment for substance use disorders, and how this in turn led to discrimination in care and hindered their access to treatment for comorbidities. For example, “Jeffrey”, a 51‐year‐old white man on methadone maintenance treatment for opioid use disorder described receiving inadequate post‐surgery back care by his physician as a result of discrimination based on drug use:

I just feel like the fact that I'm in active addiction makes it that he's [physician] less real with me. […] I just had a big surgery on my back for having a big boil. There was a big opening on my back basically and like the bandages needed to be changed, like he just couldn't be bothered, like it was just like, “here's your increase on your methadone, can I do anything else for you?” I'm like, “well, my back.” “The nurses can help you with that and this is all you need from me?” And I'm like, “I guess so.” And he just like sluffed me off so quick, yeah, I don't like him.

Among many participants, management of chronic pain owing to nerve pain or underlying conditions (e.g. arthritis and osteoporosis) was identified as a major health concern. However, participants described experiencing discrimination on the basis of their drug use, which hindered access to effective treatment. For example, “Joe”, a 60‐year‐old white man, described:

They [physicians] automatically figure that you're either seeking drugs, you know, for something that isn't there. That's my interpretation. It's just it's difficult to get something for pain if you're an addict.

Some Indigenous participants recounted how their health care experiences were tied to their interlocking social positions. For example, these participants commonly described how pervasive discrimination on the basis of drug use, race and Indigeneity by providers precluded them from accessing effective pain treatment. They explained how physicians often denied them access to opioids for pain management as a result of false beliefs and biases against Indigenous people who use drugs. As “Mark”, a 55‐year‐old Indigenous man, described:

The doctors being so difficult in trying to prescribe you. Right away they look at you as an addict, so that's the first thing they talk about – let's talk about putting down your meds a little bit. That's the first thing they'll talk about. […] I look First Nation too, that plays a role in it immediately.

### 3.3 Challenges of managing HIV alongside comorbidities

Participant accounts elucidated the challenges of juggling the management of HIV with comorbidities. Participants across the sample reported feeling overwhelmed and burdened by the management of multiple health conditions, as Lisa illustrated: “*It's a burden, it's a nuisance. I wish I didn't have these other* [non‐HIV] *ailments*”. Most participants described how they were generally well engaged in HIV care, keeping their routine medical appointments to monitor their CD4 counts and viral loads, among other clinical outcomes, and adhering to their ART regimens. However, participant narratives revealed how comorbidities were often unmanaged or poorly managed, and how symptoms from comorbidities or side effects from treatment for these conditions could adversely affect their HIV management. For example, Jeffrey recounted how treatment disruption for his opioid use disorder led to poor ART adherence. After being involuntarily cut off from methadone maintenance treatment, he stopped going to the Dr. Peter Centre, where he took his HIV medications:

Interviewer: When you described going off of the meds [ARTs] for a couple months, what was going on for you at that time?
Jeffrey: I'm not sure exactly what was going on at the time. It's when I got off, when I got kicked off the methadone, I kind of […] stopped coming here [Dr. Peter Centre] for a while. I take my meds here […] so if I'm not coming here, then I wasn't taking them.

Jeffrey also described the difficulties of keeping his appointments with unmanaged substance use disorder:

Jeffrey: I've missed lots of appointments, yeah. It's just, my concentration is somewhere else, I guess.
Interviewer: Where is your concentration?
Jeffrey: Well, it's in active addiction.

Lisa explained how taking benzodiazepines to manage an anxiety disorder induced fatigue and sometimes limited her ability to keep her appointments:

The Benzos have a lot to do with my fatigue. And sometimes I do miss appointments cause I'm tired. Or I just don't want to get up.

These challenges experienced by participants with regard to managing HIV alongside comorbidities suggest lack of coordination in their care. Participant narratives highlighted how they not only live with the burden of their illnesses, but also the burden of managing their care for multiple, concurrent conditions.

### 3.4 Concerns regarding impact of ART on comorbidities

For some participants, the potential impact of ART on the development of comorbidities was a source of concern. While most participants described how they continued to take ART, several participants expressed apprehensions about the drugs’ cumulative effects on various organs, including the kidney, liver and heart, and resulting morbidity and mortality. As “Larry”, a 68‐year‐old Asian man described:

I wonder after like 25 years of taking an HIV med, is it hard on my liver and kidney? Cause obviously the drug has some toxin. […] I don't think the HIV will kill me, I think more likely other issues like maybe kidney failure or liver failure or heart, or maybe heart attack.

For Mark, concerns over the possible contribution of ART to the development of comorbidities made him reluctant to make any changes to his current ART regimen. He lives with COPD and explained the dilemma of having to weigh the option of an alternative HIV treatment, which may improve his immune system and lung function, but bring with it a new risk for liver damage:

They [physicians] want to start me on a new treatment [for HIV] to see if my COPD can go away with that new med, but I'm refusing to take it because it says something to do with damaging your liver, so I thought why should I take a drug that is going to damage the liver when my liver is fine right now, so just weighing out options. That's where I'm at, at the moment.

Mark later discussed how he would not take ART if it were to increase his risk of developing new comorbidities and cause more harm:

I've been going on like this for decades and I'm at a point now, I want to continue fighting to survive health‐wise, and if HIV meds are helping, wonderful, but if they're going to do damage like I said, then I don't want to pursue it, I'll just let the virus take its toll.

For some participants, concerns over the impact of ART on comorbidities were framed by past experiences with early, toxic ART regimens. “Chris”, a 50‐year‐old white man, described how his previous ART regimen led to restless leg syndrome and nerve damage in his legs and feet, which prevented him from walking. After switching to a new regimen, he has been able to walk but still takes gabapentin to manage his nerve pain:

The other health conditions like the restless leg syndrome and the nerve damage in my legs and feet are from one of the other cocktails that I was on. […] One of the side effects from another HIV cocktail that I was on. I have to take Gabapentin every day. So, yeah, my legs still hurt but they don't twitch as much as they used to. I spent nine years in an electric wheelchair. Now I walk.

For “Paul”, a 60‐year‐old white man, trepidations about how ART might lead to comorbidities were rooted in previous experiences with toxic ART regimen alongside negative health care encounters. Paul recounted how he was unwillingly put on AZT (zidovudine) by his physician, which he believed led to a compromised immune system, and ultimately, development of progressive multifocal leukoencephalopathy (PML), a severe viral infection of the brain:

I got anemia and my doctor said, 'Oh, no, it's HIV‐related.' It wasn't HIV. It was a viral anemia. He says, 'But it doesn't matter, you still have to go on viral medications. You have to go on the HIV medications.' I'm, like, 'Yeah, okay. What the hell, I don't know what I'm doing.' And then my immune system crashed. Everything went nuts and I almost died from taking too much medication. The AZT, whatever it was, HIV medication had a bad reaction and I came out delusional. […] And then it was later discovered […] that I had PML, it was a brain infection that nearly killed me.

### 3.5 Integrated approaches to care

Many participants expressed the importance of care based on integrated approaches that address the multitude of health and psychosocial issues that they experience. Several received care at a local primary care clinic for PLHIV and explained how their various needs were met through the clinic's integrated services model that provided holistic care, as “Robert”, a 62‐year‐old white man, described:

It [comprehensive primary care clinic] is a good place, you know. They're really good there. You know how you go to the doctor. You can only see him one symptom at a time. Well, with us, it's a lot different because a lot of things happen to us, right. And so, they don't care about that. You know, you maybe go in with multiple different things and they'll fix you up. […] You know, nutrition, you know, you can go there and they do your blood work. They have counselors there if you need them. Just everything in general.

Participants described how care coordination and co‐location of services facilitated greater ease and confidence in engaging in the concurrent management of their HIV and comorbidities. For example, care coordination allowed participants to keep their medical appointments. Jamie, a resident of the Dr. Peter Centre, explained how care coordination, including in‐person reminders by the centre's nurses, facilitated his ability to keep his HIV and non‐HIV‐related clinic appointments:

They help me, the staff [at Dr. Peter Centre], to remind me because I have so many appointments. The eye doctor, hearing aids, heart. […] My family physician, my HIV doctor. So, I had a lot of appointments there for a while. Five days a week. Nonstop all day. So, I made sure I kept them.

Participants whose ART was co‐administered with medication for comorbidities, such as methadone, described how the daily, coordinated dispensing and witnessed ingestion, typically at pharmacies or the Dr. Peter Centre, encouraged their treatment adherence. “Jim”, a 60‐year‐old white man who reported being cured of hepatitis C, recounted how integrated approach to dispensation of his medications, including hepatitis C treatments, ART and methadone, made the management of multiple comorbidities simple:

Interviewer: Do you find it was difficult to manage all of those medications?
Jim: No. No. No, because we made it to where I get it every day. I had daily dispensed methadone at the time, so we had everything there in one shot. So I'd go there in the morning and take it and that was it.

Finally, clients at the Dr. Peter Centre described receiving care that was responsive to features of the risk environment (e.g. housing insecurity and food insecurity) through the centre's integrated approach to health and social services. For example, Jeffrey described receiving counselling as well as support around securing housing to address the underlying causes of his depression:

I went through depression. They [Dr. Peter Centre] would assist me in counselling and whatever is out in the community that I can access. […] They got me through a lot of my grief and there is a lot of support here. […] I go through like homelessness, they help me with letters and whatever information that they have out there that they're aware of.

Participants described feeling supported and experiencing improved wellbeing when they received support that addressed the full spectrum of their presenting needs.

## 4 DISCUSSION

Our study contributes to the nascent body of literature on comorbidities among an ageing population of PLHIV. Specifically, this study adds the perspectives of older PLHIV who use drugs, a growing but understudied population disproportionately affected by comorbidities, and their experiences with health management. Many participants prioritized comorbidities over HIV as access to effective treatment was more difficult for these conditions. Our study also revealed the everyday challenges of managing HIV alongside comorbidities, juggling multiple health needs. While motivation to engage in HIV treatment was generally high, for some participants, the potential impact of ART on the development of comorbidities was a source of concern and frustration. Participant narratives demonstrated the critical need for integrated care to support the concurrent management of HIV and comorbidities.

Perhaps unsurprisingly, given advances in ART and its universal coverage in our setting, participants in our study prioritized comorbidities over HIV. In line with our finding, previous work from the United States has identified comorbidities as a source of concern among PLHIV, in some cases overshadowing HIV‐related concerns [23]. Moreover, studies on risk perceptions of people who use drugs have suggested that more immediate concerns, such as drug withdrawals, overdoses and conditions stemming from their structural vulnerability (e.g. poverty, homelessness and incarceration), are often prioritized over more distal, less tangible health concerns, such as HIV and other injection‐related infections [24, 25, 26]. We extend existing research by identifying that comorbidities were prioritized among older PLHIV who use drugs as treatment for these conditions was challenging, or in many cases, impossible to access due to social‐structural forces operating within the risk environment, particularly stigma and discrimination in health care encounters. A large body of literature has established that stigma, including anti‐drug stigma, is pervasive and often leads to discrimination in health care settings [27, 28]. In addition to anti‐drug stigma and discrimination, we found that intersectional stigma and discrimination on the basis of drug use, race and Indigeneity hindered access to treatment for comorbidities among older PLHIV who use drugs. Recent work has raised concerns about the multiple and intersecting forms of stigma that PLHIV may experience [29, 30]. While intersectional stigma is a common experience, the effects and mechanisms through which it may contribute to adverse health are not well understood [31]. Our study demonstrates how intersectional stigma manifests as acts of discrimination in health care settings and offers emerging evidence of its harmful impacts on the health management of older PLHIV who use drugs. Future work should examine strategies to reduce stigma and discrimination within the health care system and at the population level, including but not limited to structural competency training for health care providers and decriminalization of drugs, in order to ensure improved and equitable access to comorbidities care of this population.

Our research illustrates the complexity of concurrently managing HIV and comorbidities for older PLHIV who use drugs. In line with previous research reporting that PLHIV worry about the potential adverse effects of ART [32, 33], participant narratives demonstrated concerns over ART and its possible role in increasing the risk for comorbidities. For some of our participants, these concerns were shaped by past experiences with toxic ART regimens as well as health care encounters where they felt that their health experiences were not heard or taken into consideration. Previous studies have suggested that the perceived level of control that PLHIV have over decisions about their treatment is important for ART uptake [33]. Our findings highlight the critical need for effective communication between health care professionals and patients to ensure shared decision‐making regarding treatments. Patient‐centred approaches to care that respect patient concerns, needs and preferences should be adopted particularly for medically complex individuals like older PLHIV who use drugs to ensure that treatment plans optimize their health and wellbeing.

Integrated care has been increasingly recognized in recent years as a necessary approach to organizing care for various populations with complex care needs, including the general older adult population and PLHIV [34, 35]. We extend current knowledge on integrated care by offering an analysis of the real‐world application of various approaches to integrated care utilized by older PLHIV who use drugs and their role in comorbidities management. In line with previous research on integrated HIV care, we found that care coordination and co‐location of services (e.g. daily dispensing and witnessed ingestion of ART and methadone) were pivotal in facilitating engagement in care for HIV alongside comorbidities among our study population [36, 37]. Furthermore, we found that integrated care improved participants’ wellbeing by addressing the full range of individuals’ needs. This is particularly important as the adverse effects of comorbidities on quality of life and wellbeing continues to be a growing concern. Our work offers further evidence to support the allocation of funding and resources needed to implement and scale‐up integrated HIV care services.

This study has limitations. First, interviews were limited to English‐speaking individuals. Thus, the experiences of individuals who are particularly marginalized in mainstream systems of care, such as new immigrants and refugees, may not have been captured. Second, many participants had been diagnosed with HIV in the 1990s or early 2000s, and thus have experiences with older HIV treatment regimens. Therefore, their experiences may not be reflective of individuals who were diagnosed more recently. Finally, most participants had long‐term HIV treatment histories as our study was conducted in a setting with longstanding universal coverage of ART and other treatment supports (e.g. directly observed therapy) for PLHIV. Research conducted in settings without these supports may reveal other challenges to managing HIV alongside comorbidities.

## 5 CONCLUSIONS

The current study advances understanding of challenges encountered by older PLHIV who use drugs and how they manage their comorbidities. Our findings underscore the need for HIV care to shift from a primary focus on managing HIV to providing integrated, patient‐centred care that addresses both HIV and non‐HIV‐related needs as well as an equitable and non‐judgemental delivery of such care for older PLHIV who use drugs.

## FUNDING

This work is supported by the National Institutes of Health [R01DA043408]. KLC is supported by a Mitacs Accelerate Fellowship. WS is supported by an Michael Smith Foundation for Health Research (MSFHR) Scholar Award. AI is supported by a Mitacs Elevate Postdoctoral Fellowship. RM is supported by an MSFHR Scholar Award and Canadian Institutes of Health Research (CIHR) New Investigator Award.

## COMPETING INTERESTS

The authors have no competing interests to declare that are relevant to the content of this article.

## AUTHORS’ CONTRIBUTIONS

KLC, CN and RM developed the study protocol. KLC and CN conducted the interviews. KLC, CN and RM developed the coding framework. KLC and CN coded the data. KLC wrote the original draft and revised it following feedback from CN, WS, AI and RM. All authors read and approved the final manuscript.
